# Early menopause is associated with increased risk of retinal vascular occlusions: a nationwide cohort study

**DOI:** 10.1038/s41598-022-10088-0

**Published:** 2022-04-12

**Authors:** Sungsoon Hwang, Se Woong Kang, Kyung Jun Choi, Ki Young Son, Dong Hui Lim, Dong Wook Shin, DooSeok Choi, Sang Jin Kim

**Affiliations:** 1 Department of Ophthalmology, Samsung Medical Center, Sungkyunkwan University School of Medicine, #81 Irwon-ro, Gangnam-gu, Seoul, 06351 Republic of Korea; 2 Department of Clinical Research Design and Evaluation, Samsung Advanced Institute for Health Sciences and Technology (SAIHST), Sungkyunkwan University, Seoul, Republic of Korea; 3 Department of Ophthalmology, Gangneung Asan Hospital, University of Ulsan College of Medicine, Gangneung, Republic of Korea; 4 Department of Family Medicine and Supportive Care Center, Samsung Medical Center, Sungkyunkwan University School of Medicine, Seoul, Republic of Korea; 5 Department of Obstetrics and Gynecology, Samsung Medical Center, Sungkyunkwan University School of Medicine, Seoul, Republic of Korea

**Keywords:** Retinal diseases, Epidemiology

## Abstract

This nationwide population-based cohort study evaluated the association between female reproductive factors and the incidence of retinal vein occlusion (RVO) and retinal artery occlusion (RAO) using data provided by the Korea National Health Insurance Service. A total of 2,289,347 postmenopausal women over 50 years of age who participated in both national health screening and cancer screening in 2013 or 2014 were included. Data on female reproductive factors, including age at menarche, age at menopause, parity, history of hormone replacement therapy, and oral contraceptive pill usage, were collected. Patients were followed up until December 2018, and incident cases of RVO and RAO were identified using registered diagnostic codes from claim data. During an average follow-up period of 4.90 years, 7461 and 1603 patients were newly diagnosed with RVO and RAO, respectively. In the multivariable-adjusted Cox proportional hazard model, patients who experienced menopause after 55 years of age had a lower risk of RVO and RAO development compared to those who had menopause before 45 years of age, with a hazard ratio (95% confidence interval) of 0.83 (0.76–0.95) for RVO and 0.80 (0.66‒0.98) for RAO. In conclusion, early menopause was an independent risk factor for future development of RVO and RAO.

## Introduction

Retinal vein occlusion (RVO) and retinal artery occlusion (RAO) are the major vision-threatening retinal vascular diseases^1,2^. The estimated annual incidences of RVO and RAO are 15 and 2 per 100,000 people, respectively^3,4^, and a recent meta-analysis estimated that RVO is present in more than 25 million individuals worldwide^5^. Both RVO and RAO occur more commonly in the older population (aged over 50 years)^6,7^, which implies an increase in healthcare-related socioeconomic burden due to retinal vascular occlusions, because of the aging of the global population.

The primary mechanism underlying RVO development is venous compression at the arteriovenous crossing site^8^, which could be further exacerbated by increased arterial rigidity arising from aging and atherosclerosis^9^. Venous compression causes turbulence in the retinal vein and subsequent endothelial damage, further leading to thrombus formation^10^. On the other hand, RAO is caused by the impaction of emboli in the retinal artery, which typically originate from atherosclerotic plaques in the carotid artery or from the cardiac valves or chambers^2^. Both RVO and RAO are associated and share common risk factors with cardiovascular diseases^11,12^. Numerous studies have established sound epidemiological evidence on the association of cardiovascular risk factors, such as hypertension, diabetes, dyslipidemia, and of cardiovascular accidents per se, including stroke and ischemic heart disease, with the risk of RVO and RAO development^13^.

Female reproductive parameters, including age at menarche/menopause, parity, and use of hormone replacement therapy (HRT), are factors that influence cardiovascular events in postmenopausal women^14,15^. However, little is known about the relevance of such reproductive factors to retinal vascular occlusions, and no study to date has comprehensively evaluated the impact of female reproductive factors on the development of RVO and RAO. In this study, we investigated the association between female reproductive factors and the risk of RVO and RAO in postmenopausal women, using a nationally representative health screening cohort from South Korea.

## Methods

### Setting

This was a nationwide, population-based, retrospective cohort study using data provided by the Korea National Health Insurance Service (NHIS). The study adhered to the tenets of the Declaration of Helsinki and was approved by the Institutional Review Board of Samsung Medical Center, Seoul, Republic of Korea (IRB File Number 2020-11-075). The Institutional Review Board of Samsung Medical Center waived the requirement for informed consent based on the use of de-identified public data and the retrospective design of the study.

In South Korea, the NHIS is the insurer that provides mandatory universal medical care and holds all medical information and health claims data. The NHIS provides National Health Screening^16^, a free biennial general health examination offered to all Koreans over 40 years of age. The NHIS also runs the National Cancer Screening program as an extension of the National Health Screening^17^. The National Cancer Screening provides examinations for the stomach, liver, colorectal, breast, and cervical cancer for all registered individuals at the age indicated for cancer screening by the government (e.g., biennial breast cancer examination starting at the age of 40 years).

The NHIS hold the data of the entire population in terms of demographic information (e.g., age, sex, income, death) and health claims data, including the date of clinical visits, prescription records, and diagnostic codes defined by the Korean Classification of Diseases 7th revision (KCD-7), which is based on the International Classification of Diseases, 10th revision, but with a few changes specific to Korea. The NHIS also holds data from the National Health Screening and National Cancer Screening (e.g., questionnaire responses, anthropometric measurements, and laboratory test results), which can be linked to the health claims data through de-identified key numbers assigned to individuals. This database has been widely used in previous studies that have identified associations between various diseases and risk factors^18–20^. Detailed database profile information is provided elsewhere^16,17^.

### Subjects

**Figure 1:**
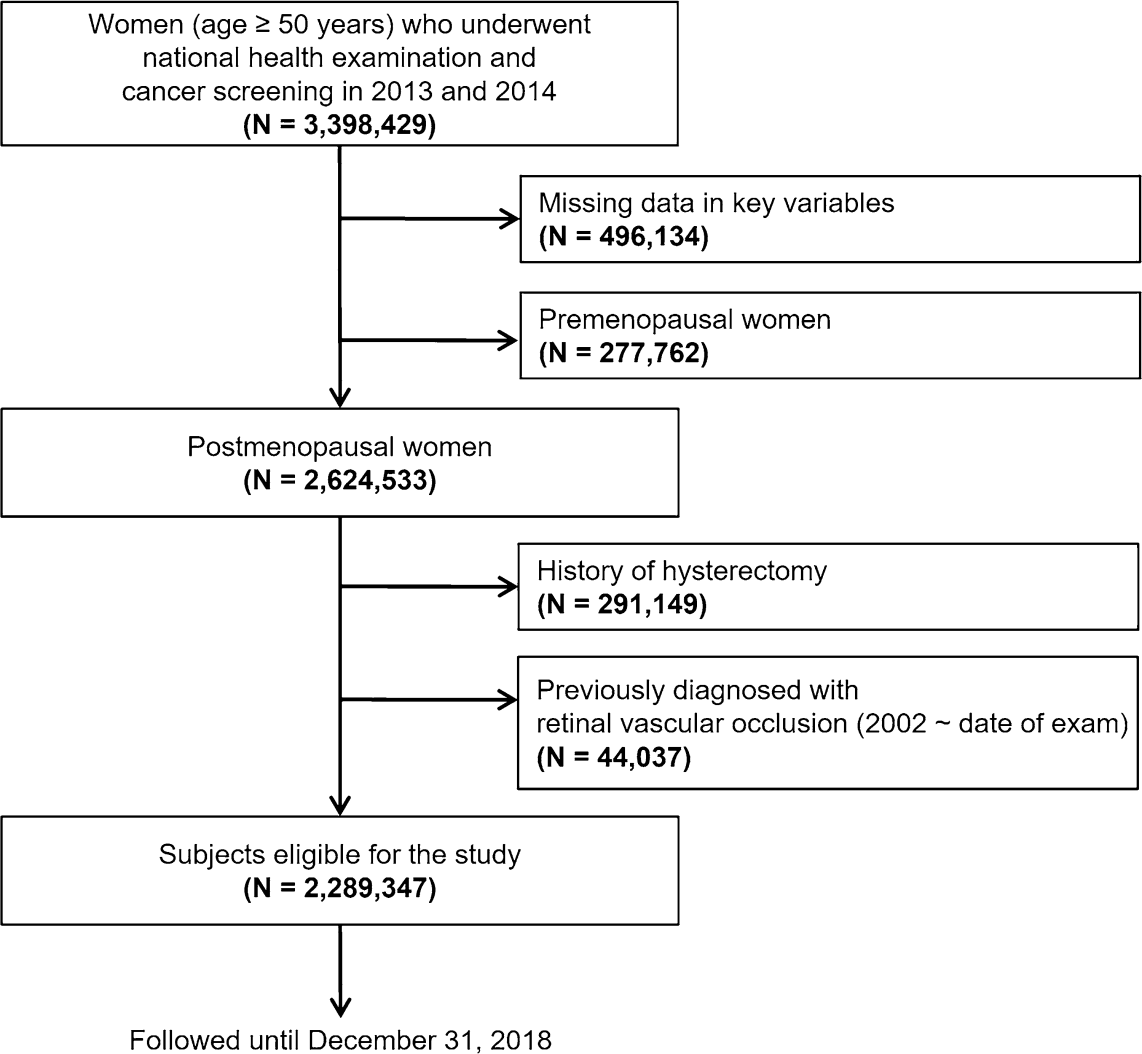
Flow chart of the cohort study design.

Of the 3,398,429 female subjects over 50 years of age who participated in both the National Health Screening and National Cancer Screening (for breast cancer) in 2013 or 2014, a total of 2,624,533 eligible postmenopausal women without missing key information were identified. Key information included response to questionnaire including history of hypertension, diabetes, dyslipidemia, stroke, and heart disease, response to behavioral factors item, response to reproductive factors item, body mass index (BMI), and lab result of blood creatinine level. Individuals who had a history of hysterectomy (n = 291,149) were excluded from the study, because they could possibly have confounding reproductive health issues, which would disturb patients' exposure to endogenous female hormones. Individuals diagnosed with retinal vascular occlusions (KCD-7 code: H34) before the examination (n = 44,037) were also excluded, because the study outcome focused on incident cases of RVO and RAO that developed after the examination. Finally, 2,289,347 postmenopausal women were included in the study (Fig. 1).

### Systemic comorbidities, behavioral factors, and female reproductive factors

Comorbid hypertension, diabetes, and dyslipidemia were identified based on self-reported questionnaire responses, health screening measurement results of blood pressure (hypertension, systolic blood pressure ≥ 140 mmHg or diastolic blood pressure ≥ 90 mmHg); fasting glucose (diabetes, fasting blood glucose levels ≥ 126 mg/dL); total cholesterol (dyslipidemia, ≥ 240 mg/dL); and the presence of diagnostic codes (KCD-7 code: I15 for hypertension, E11–E14 for diabetes, E78 for dyslipidemia) combined with medication prescription codes within a year before the health screening examination. Chronic kidney disease was defined as an estimated glomerular filtration rate of < 60 ml/min/1.73 m^2^ calculated from serum creatinine level. Income level was categorized into quartiles according to the insurance premium level, which was determined by the total household income.

Data regarding health-related behaviors were collected using the participants' responses to the health screening questionnaire. Smoking status was used to classify participants as non-, past-, or current smokers. Drinking habits were categorized as none, mild (< 30 g of alcohol/day), or heavy (≥ 30 g/day). Regular exercise was defined as performing a moderate level of physical activity for more than 30 min per day for more than 5 days per week. BMI was calculated as the weight (kg) divided by height squared (m^2^), and categorized as underweight (BMI < 18.5 kg/m^2^), normal weight (18.5 ≤ BMI < 23 kg/m^2^), overweight (23 ≤ BMI < 25 kg/m^2^), obese I (25 ≤ BMI < 30 kg/m^2^), and obese II (≥ 30 kg/m^2^), according to the Korean Society for the Study of Obesity^21^.

Reproductive parameters were retrieved from the responses to the cancer screening questionnaire. The questionnaire items included age at menarche and menopause, parity, total lifetime breastfeeding, duration of HRT, and oral contraceptive pill use. Data on female reproductive factors were categorized as follows: age at menarche (< 13 years, 13–14 years, 15–16 years, and ≥ 17 years); age at menopause (< 40 years, 40–44 years, 45–49 years, 50–54 years, and ≥ 55 years), parity (0, 1, ≥ 2 children); lifetime breastfeeding history (never, < 6 months, 6 to < 12 months, and ≥ 12 months); duration of HRT (never, < 2 years, 2 to < 5 years, ≥ 5 years, and unknown); and duration of oral contraceptive pill use (never, < 1 year, ≥ 1 year, unknown).

### Identification of retinal vascular occlusions and follow-up

RVO cases were defined as having two or more medical claims with diagnostic codes for RVO (KCD-7 code: H34.8) on different dates, and the date of the first diagnostic code for RVO was regarded as the incident time of RVO. RAO cases were defined as having two or more medical claims with diagnostic codes for RAO (KCD-7 code: H34.1, H34.2) on different dates, and the date of the first diagnostic code for RAO was regarded as the incident time of RAO. Patients were followed from the date of health check-up to the date of incident RVO/RAO, death, or the end of the study period (December 31, 2018), whichever came first.

### Statistical analyses

The incidence rates of RVO and RAO were calculated by dividing the number of incident cases by the total number of person-years. We used Cox proportional hazard models to estimate hazard ratios (HRs) and 95% confidence intervals (CIs). In each Cox model, we adjusted for certain variables, as follows: model 1, age; model 2, age, income level, systemic comorbidities (hypertension, diabetes, dyslipidemia, stroke, heart disease, and chronic kidney disease), and behavioral factors (smoking status, drinking habit, regular exercise, and BMI); mode 3, age, income level, systemic comorbidities, behavioral factors, and female reproductive factors (age at menarche, age at menopause, parity, duration of breastfeeding, duration of HRT, and duration of oral contraceptive pill use). Age was handled as a continuous variable in Cox models. The assumption of proportional hazard was tested using scaled Schoenfeld residuals chart. The plots of Schoenfeld residuals against time did not show any pattern of changing residuals for every covariate included in the analyses. Restricted cubic spline curves of the adjusted HR and 95% CI for incident RVO and RAO according to relevant covariates were obtained with reference point at 5th percentile and knots at 5th, 35th, 65th, and 95th percentile of distribution. All statistical analyses were performed using SAS version 9.4 (SAS Institute Inc., Cary, NC, USA) and R statistical software version 4.0.4 (R Project for Statistical Computing). P-values were two-sided and considered statistically significant at values less than 0.05.

## Results

### Baseline characteristics

Table 1 presents the detailed baseline characteristics of the study participants. The mean age of the study participants was 62.41 years. The mean age at menarche and at menopause was 16.19 and 50.63 years, respectively. Of the participants, 5.28% experienced menopause before 45 years of age, and 15.71% of subjects reported that they had previously received HRT. The average follow-up period was 4.90 years, and 7461 and 1603 patients were newly diagnosed with RVO and RAO, respectively, during the follow-up period.

**Table 1:** Baseline characteristics of the study population.

Variables	Total (N = 2,289,347)
**1. Demographic factors**	
Age, years, mean ± SD	62.41 ± 8.25
Age group, No. (%)	
50–54 years	471,472 (20.59)
55–59 years	496,721 (21.70)
60–64 years	514,585 (22.48)
65–69 years	291,718 (12.74)
70–74 years	310,752 (13.57)
75–79 years	125,953 (5.50)
≥ 80 years	78,146 (3.41)
Income, No. (%)	
Q1 (lowest)	520,295 (22.73)
Q2	404,520 (17.67)
Q3	540,526 (23.61)
Q4 (highest)	824,006 (35.99)
**2. Systemic comorbidities**	
Hypertension, No. (%)	
No	1,331,201 (58.15)
Yes	958,146 (41.85)
Diabetes mellitus, No. (%)	
No	1,955,305 (85.41)
Yes	334,042 (14.59)
Dyslipidemia, No. (%)	
No	1,624,088 (70.94)
Yes	665,259 (29.06)
Stroke, No. (%)	
No	2,253,049 (98.41)
Yes	36,298 (1.59)
Heart diseases, No. (%)	
No	2,196,562 (95.95)
Yes	92,785 (4.05)
Chronic kidney disease, No. (%)	
No	2,100,209 (91.74)
Yes	189,138 (8.26)
**3. Behavioral factors**	
Smoking history, No. (%)	
Never smoked	2,210,674 (96.56)
Former smoker	25,971 (1.13)
Current smoker	52,702 (2.30)
Drinking habit, No. (%)	
None	1,987,784 (86.83)
Mild	283,589 (12.39)
Heavy	17,974 (0.79)
Regular physical activity, No. (%)	
No	1,796,638 (78.48)
Yes	492,709 (21.52)
Body mass index, No (%)	
< 18.5 kg/m^2^	54,225 (2.37)
18.5 to < 23 kg/m^2^	840,944 (36.73)
23 to < 25 kg/m^2^	590,348 (25.79)
25 to < 30 kg/m^2^	705,413 (30.81)
≥ 30 kg/m^2^	98,417 (4.30)
**4. Reproductive factors**	
Age at menarche, mean ± SD	16.19 ± 2.02
Age at menarche in group, No. (%)	
< 14 years	142,577 (6.23)
14–15 years	734,166 (32.07)
16–17 years	875,342 (38.24)
≥ 18 years	537,262 (23.47)
Age at menopause, mean ± SD	50.63 ± 3.91
Age at menopause in group, No. (%)	
< 45 years	120,874 (5.28)
45–49 years	529,786 (23.14)
50–54 years	1,333,631 (58.25)
≥ 55 years	305,056 (13.33)
Parity, No. (%)	
Nulliparous	40,211 (1.76)
1 child	186,256 (8.14)
≥ 2 children	2,062,880 (90.11)
Duration of breastfeeding, No. (%)	
Never	191,413 (8.36)
< 0.5 year	208,816 (9.12)
0.5 to < 1 year	426,428 (18.63)
≥ 1 year	1,462,690 (63.89)
Hormone replacement therapy, No. (%)	
Never used	1,834,474 (80.13)
< 2 years	202,796 (8.86)
2 to < 5 years	86,281 (3.77)
≥ 5 years	70,579 (3.08)
Unknown	95,217 (4.16)
Oral contraceptive pill use, No. (%)	
Never used	1,838,861 (80.32)
< 1 year	201,721 (8.81)
≥ 1 year	134,661 (5.88)
Unknown	114,104 (4.98)

SD, standard deviation; Q, quartile.

Supplemental Table 1 shows the baseline parameters of patients with and without incident RVO and those with and without incident RAO during the follow-up period. The mean age at baseline was greater for RVO and RAO cases than for non-RVO and non-RAO cases. RVO and RAO cases also had a greater prevalence of systemic comorbidities.

### Female reproductive factors and retinal vascular occlusions

**Figure 2:**
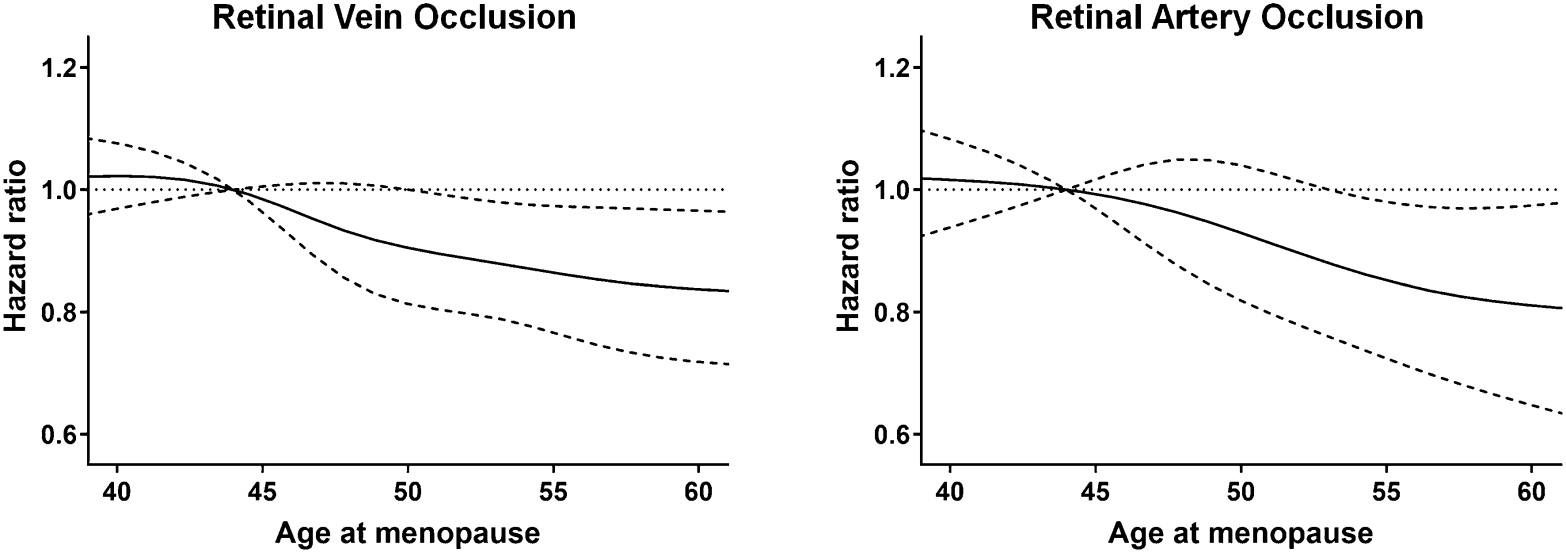
Restricted cubic spline curves presenting the adjusted hazard ratio for incident retinal vein occlusion and retinal artery occlusion according to age at menopause. Solid lines represent hazard ratio and dashed lines indicate 95% confidence interval based on restricted cubic splines for age at menopause with knots at the 5th, 35th, 65th, and 95th percentiles and reference point at the 5th percentiles of the distribution. The risk of retinal vein occlusion and retinal artery occlusion continuously decreased as age at menopause increased in Cox proportional hazard model fully adjusted for age, income level, systemic comorbidities, behavioral factors, and female reproductive factors (model 3).

Table 2 shows the incidence rates and HRs with 95% CIs of RVO development according to various reproductive factors in postmenopausal women. The overall incidence rate of RVO was 66.66 per 100,000 person-years. In model 3, the HR (95% CI) for RVO was 0.85 (0.76‒0.95), 0.88 (0.80‒0.96), and 0.90 (0.82‒0.99) for subjects aged ≥ 55 years, 50–54 years, and 45–49 years at menopause, respectively, as compared to those aged < 45 years at menopause. Accordingly, early menopause was associated with a greater risk of RVO development. A history of HRT and oral contraceptive pill use did not significantly affect the risk of developing RVO. Table 3 presents the incidence rates and HRs with 95% CI of incident RAO according to reproductive parameters. The overall incidence rate of RAO was 14.30 per 100,000 person-years. Patients who experienced early menopause (age < 45 years) had an incidence rate of 19.46 per 100,000 person-years. In Cox model 3, the HR (95% CI) for RAO was 0.80 (0.66–0.98) for those aged ≥ 55 years at menopause, as compared to those aged < 45 years at menopause. Other reproductive parameters were not significantly associated with risk of developing RAO. Figure 2 demonstrates restricted cubic spline models for the association between age at menopause and the risk of incident RVO and RAO. The risks of RVO and RAO continuously decrease as age at menopause increases.

**Table 2:** Hazard ratios and 95% confidence intervals for development of retinal vein occlusion in postmenopausal women according to female reproductive factors.

Retinal vein occlusion	Subject no.	Case no.	Duration (person-years)	IR per 100,000 person-years	Model 1 HR (95% CI)	Model 2 HR (95% CI)	Model 3 HR (95% CI)
**Overall**	2,289,347	7461	11,192,885	66.66			
Age at menarche							
< 14 years	142,577	344	690,223	49.84	1.00 (ref)	1.00 (ref)	1.00 (ref)
14–15 years	734,166	2151	3,569,702	60.26	1.09 (0.97–1.23)	1.10 (0.98–1.23)	1.10 (0.98–1.23)
16–17 years	875,342	2891	4,289,549	67.40	1.08 (0.96–1.21)	1.08 (0.96–1.21)	1.07 (0.95–1.20)
≥ 18 years	537,262	2075	2,643,411	78.50	1.13 (1.00–1.27)	1.14 (1.01–1.28)	1.12 (0.99–1.26)
Age at menopause							
< 45 years	120,874	534	595,118	89.73	1.00 (ref)	1.00 (ref)	1.00 (ref)
45–49 years	529,786	1782	2,597,852	68.60	0.88 (0.80–0.97)	0.89 (0.81–0.98)	0.90 (0.82–0.99)
50–54 years	1,333,631	4098	6,509,463	62.95	0.85 (0.78–0.93)	0.86 (0.79–0.94)	0.88 (0.80–0.96)
≥ 55 years	305,056	1047	1,490,454	70.25	0.85 (0.76–0.94)	0.84 (0.76–0.94)	0.85 (0.76–0.95)
Parity							
Nulliparous	40,211	130	194,953	66.68	1.00 (ref)	1.00 (ref)	1.00 (ref)
1 child	186,256	518	904,174	57.29	0.94 (0.78–1.14)	0.95 (0.79–1.16)	0.96 (0.78–1.18)
≥ 2 children	2,062,880	6813	10,093,759	67.50	0.90 (0.75–1.07)	0.91 (0.76–1.08)	0.89 (0.74–1.09)
Duration of breastfeeding							
Never	191,413	528	925,916	57.02	1.00 (ref)	1.00 (ref)	1.00 (ref)
< 0.5 year	208,816	488	1,006,405	48.49	0.88 (0.78–0.99)	0.90 (0.80–1.02)	0.93 (0.81–1.06)
0.5 to < 1 year	426,428	1123	2,079,940	53.99	0.89 (0.80–0.99)	0.90 (0.81–1.00)	0.94 (0.84–1.05)
≥ 1 year	1,462,690	5322	7,180,625	74.12	1.01 (0.92–1.11)	1.01 (0.92–1.11)	1.04 (0.94–1.15)
Hormone replacement therapy							
Never used	1,834,474	5995	8,966,668	66.86	1.00 (ref)	1.00 (ref)	1.00 (ref)
< 2 years	202,796	620	995,995	62.25	1.07 (0.98–1.16)	1.08 (0.99–1.17)	1.07 (0.99–1.17)
2–5 years	86,281	286	424,267	67.41	1.09 (0.96–1.23)	1.11 (0.98–1.25)	1.11 (0.98–1.25)
≥ 5 years	70,579	266	347,636	76.52	1.13 (0.99–1.28)	1.14 (1.00–1.29)	1.13 (0.99–1.28)
Unknown	95,217	294	458,319	64.15	0.91 (0.81–1.03)	0.9 (0.8–1.01)	0.93 (0.82–1.06)
Oral contraceptive pill use							
Never used	1,838,861	5919	8,989,145	65.85	1.00 (ref)	1.00 (ref)	1.00 (ref)
< 1 year	201,721	682	989,036	68.96	1.07 (0.98–1.15)	1.05 (0.97–1.14)	1.05 (0.97–1.13)
≥ 1 year	134,661	501	662,606	75.61	1.09 (1.00–1.20)	1.05 (0.96–1.16)	1.04 (0.95–1.14)
Unknown	114,104	359	552,098	65.02	0.93 (0.83–1.03)	0.91 (0.82–1.02)	0.95 (0.85–1.06)

Model 1: adjusted for age. Model 2: adjusted for age, income level, systemic comorbidities (hypertension, diabetes mellitus, dyslipidemia, stroke, heart disease, and chronic kidney disease), and behavioral factors (smoking history, drinking habit, physical activity, body mass index). Model 3: adjusted for age, income level, systemic comorbidities, behavioral factors, and female reproductive factors (age at menarche, age at menopause, parity, breastfeeding, hormone replacement therapy, oral contraceptive pill).

IR, incidence rate; HR, hazard ratio; CI, confidence interval.

**Table 3:** Hazard ratios and 95% confidence intervals for development of retinal artery occlusion in postmenopausal women according to female reproductive factors.

Retinal artery occlusion	Subject no.	Case no.	Duration (person-years)	IR per 100,000 person-years	Model 1 HR (95% CI)	Model 2 HR (95% CI)	Model 3 HR (95% CI)
**Overall**	2,289,347	1603	11,207,750	14.30			
Age at menarche							
< 14 years	142,577	87	690,823	12.59	1.00 (ref)	1.00 (ref)	1.00 (ref)
14–15 years	734,166	435	3,574,104	12.17	0.86 (0.69–1.09)	0.87 (0.69–1.09)	0.87 (0.69–1.10)
16–17 years	875,342	651	4,295,258	15.16	0.92 (0.73–1.15)	0.92 (0.73–1.15)	0.93 (0.74–1.16)
≥ 18 years	537,262	430	2,647,565	16.24	0.88 (0.70–1.12)	0.89 (0.71–1.13)	0.90 (0.71–1.14)
Age at menopause							
< 45 years	120,874	116	596,210	19.46	1.00 (ref)	1.00 (ref)	1.00 (ref)
45–49 years	529,786	409	2,601,311	15.72	0.96 (0.81–1.16)	0.97 (0.82–1.17)	0.97 (0.82–1.17)
50–54 years	1,333,631	869	6,517,655	13.33	0.87 (0.75–1.03)	0.89 (0.76–1.05)	0.88 (0.76–1.05)
≥ 55 years	305,056	209	1,492,575	14.00	0.81 (0.67–0.99)	0.80 (0.67–0.98)	0.80 (0.66–0.98)
Parity							
Nulliparous	40,211	15	195,240	7.68	1.00 (ref)	1.00 (ref)	1.00 (ref)
1 child	186,256	101	905,200	11.16	1.61 (0.94–2.77)	1.64 (0.95–2.82)	1.53 (0.87–2.69)
≥ 2 children	2,062,880	1487	10,107,311	14.71	1.59 (0.96–2.65)	1.62 (0.97–2.70)	1.52 (0.89–2.62)
Duration of breastfeeding							
Never	191,413	90	926,977	9.71	1.00 (ref)	1.00 (ref)	1.00 (ref)
< 0.5 year	208,816	114	1,007,344	11.32	1.20 (0.91–1.58)	1.24 (0.94–1.63)	1.16 (0.87–1.54)
0.5 to < 1 year	426,428	270	2,082,088	12.97	1.21 (0.95–1.53)	1.23 (0.97–1.57)	1.16 (0.90–1.50)
≥ 1 year	1,462,690	1129	7,191,342	15.70	1.14 (0.92–1.42)	1.14 (0.92–1.42)	1.07 (0.84–1.36)
Hormone replacement therapy							
Never used	1,834,474	1294	8,978,530	14.41	1.00 (ref)	1.00 (ref)	1.00 (ref)
< 2 years	202,796	120	997,324	12.03	1.00 (0.83–1.21)	1.00 (0.83–1.21)	1.00 (0.83–1.21)
2–5 years	86,281	65	424,809	15.30	1.23 (0.95–1.59)	1.24 (0.96–1.60)	1.24 (0.96–1.61)
≥ 5 years	70,579	51	348,194	14.65	1.08 (0.82–1.43)	1.08 (0.82–1.43)	1.08 (0.82–1.43)
Unknown	95,217	73	458,893	15.91	1.03 (0.81–1.30)	1.01 (0.80–1.28)	1.05 (0.81–1.35)
Oral contraceptive pill use							
Never used	1,838,861	1287	9,000,859	14.30	1.00 (ref)	1.00 (ref)	1.00 (ref)
< 1 year	201,721	131	990,479	13.23	0.95 (0.80–1.14)	0.94 (0.78–1.12)	0.93 (0.77–1.11)
≥ 1 year	134,661	103	663,629	15.52	1.02 (0.83–1.24)	0.96 (0.79–1.18)	0.95 (0.78–1.17)
Unknown	114,104	82	552,783	14.83	0.95 (0.76–1.19)	0.93 (0.75–1.17)	0.92 (0.72–1.17)

Model 1: adjusted for age. Model 2: adjusted for age, income level, systemic comorbidities (hypertension, diabetes mellitus, dyslipidemia, stroke, heart disease, and chronic kidney disease), and behavioral factors (smoking history, drinking habit, physical activity, body mass index). Model 3: adjusted for age, income level, systemic comorbidities, behavioral factors, and female reproductive factors (age at menarche, age at menopause, parity, breastfeeding, hormone replacement therapy, oral contraceptive pill).

IR, incidence rate; HR, hazard ratio; CI, confidence interval.

### Other factors and retinal vascular occlusions

The association between other baseline parameters and the risk of retinal vascular occlusions is demonstrated in Supplemental Table 1 (RVO) and Supplemental Table 1 (RAO). Income level was not associated with the incidence of retinal vascular occlusions. All systemic comorbidities included in the study were significantly associated with both RVO and RAO in age-adjusted model 1. Mild drinking was independently associated with a lower risk of retinal vascular occlusions than no drinking (HR [95% CI]; 0.79 [0.72–0.86] for RVO and 0.74 [0.61–0.90] for RAO).

## Discussion

This nationwide population-based cohort study included a large number of postmenopausal women and identified the reproductive risk factors for incident retinal vascular occlusions. Several large-scale cohort studies and meta-analyses have previously identified systemic risk factors for retinal vascular occlusion development. The representative cohort studies, including the Beaver Dam Eye Study^3,22^ from the United States and the Blue Mountains Eye Study^23,24^ from Australia, emphasized the significant impact of cardiovascular risk factors on the incidence of RVO and RAO. However, female reproductive parameters, which are significant determinants of cardiovascular diseases, were not assessed in these cohort studies or in any other studies regarding retinal vascular occlusions to date; thus, this merits further research. Adding to the previous literature, the current study provides novel information regarding the relevance of female reproductive factors to retinal vascular occlusions.

In the present study, early menopause was an independent risk factor for incident RVO and RAO. Menopause before the age of 45 years is generally defined as early menopause and occurs in approximately 5% of women worldwide^25,26^. Early menopause has been reported to be linked to incident cardiovascular events and cardiovascular disease-related mortality^27^. The main underlying mechanisms for the association between early menopause and increased risk of postmenopausal cardiovascular disease are the protective effects of endogenous estrogens on the cardiovascular system. Endogenous estrogen promotes vasodilation and inhibits the response of blood vessels to injury, thereby preventing the development of atherosclerosis^28^. Loss of these protective effects increases the expression of inflammatory cytokines that could further damage the endothelium of vessels^29^. Early menopause indicates a lower lifetime exposure to the beneficial effect of endogenous estrogen and early exposure to more significant vascular damage, leading to an increased risk of cardiovascular events. Endogenous estrogen may also influence the vascular health of the retinal artery and veins, via the same mechanism. This could contribute to the greater risk of future RVO and RAO development in patients with early menopause.

A history of HRT was not associated with the incidence of RVO or RAO. Additional subgroup analyses according to age at menopause and age at baseline failed to prove any association between a history of HRT and the incidence of retinal vascular occlusions (Supplemental Table 1). While endogenous estrogen during the reproductive period is well-known for its antioxidant and anti-inflammatory effects and is generally regarded to have a protective effect on the vascular system^30^, the effect of HRT on vascular health is complex^31^. Initially, HRT was introduced with the expectation that it would reduce the risk of cardiovascular disease. Contrary to this expectation, however, large-scale clinical trials, including the Heart and Estrogen‒Progestin Replacement Study and the Women's Health Initiative study, reported that HRT showed no preventive effect and rather increased the risk of cardiovascular diseases by 50‒80% in the first year of treatment^32,33^. According to a recent concept, the effect of HRT on blood vessels may differ depending on the timing of administration of the drug, the type of drug, and the dosage^31^. For instance, HRT can have a detrimental effect on blood vessels in individuals who have passed menopause long time ago, while it is expected to reduce the future incidence of cardiovascular diseases and all-cause mortality in a younger population^34^. This complexity is explained by the dual opposing actions of estrogen on the cardiovascular system: delaying the progression of early-stage atherosclerosis through beneficial effects on endothelial function and blood lipids, while potentially causing acute vascular events in the presence of advanced vascular lesions through pro-coagulant mechanisms^35^. Therefore, currently, it is recommended that HRT should be initiated in women aged < 60 years or who were fewer than 10 years from menopausal onset and without a history of cardiovascular diseases^36,37^. The absence of association between HRT and the risk of RVO and RAO in the present study could possibly be attributed to the heterogeneity of the timing of HRT initiation and its dosage. Unfortunately, we were unable to retrieve more detailed data regarding the age at which HRT was initiated, the time between menopause and HRT initiation, and the composition and concentration of HRT, since these issues were not addressed in the questionnaire items. A future study that could evaluate information about timing and dosage of HRT in detail would elucidate the stratified effect of HRT on the risk of RVO and RAO development.

Although not the primary variables of interest in this study, hypertension, diabetes, dyslipidemia, and a history of other cardiovascular diseases were associated with RVO and RAO development, which is in accordance with what was previously known^5^. Interestingly, the income level was not associated with the incidence of retinal vascular occlusion. People with a higher income are expected to visit hospitals more readily and receive proper management for visual deterioration, while those with a lower income may be more likely to ignore symptoms of retinal vascular occlusion^38^. However, this was not the case in the present study. Our results might be attributable to the easy hospital accessibility supported by the unique nationwide insurance coverage and financial support in South Korea. The present study also showed the protective effect of mild drinking on both RVO and RAO. Although it is still controversial, mild drinking is suggested to have protective effect on cardiovascular diseases by improving the blood lipid profile and decreasing thrombosis^39–41^. However, the epidemiological evidence for a beneficial effect of mild drinking on retinal vascular occlusions is not sufficient^42,43^. Therefore, the current study adds to the available literature in this area of research.

This study has some limitations that need to be addressed. First, we identified retinal vascular occlusions from the claim data. Therefore, medical chart level or multimodal imaging-based verification of the diseases was not possible. The study also might have missed asymptomatic retinal vascular occlusions or such occlusions in individuals who were unable to access the healthcare system. Therefore, retinal vascular occlusions in the present study should be regarded as clinically diagnosed retinal vascular occlusions. Second, female reproductive parameters were collected based on a self-reported questionnaire. Therefore, there is a possibility of bias attributable to inaccurate recall. In addition, since no formal definition of menopause was presented in the questionnaire, some people may have given inaccurate information on their age at menopause. Third, the definition of the presence of heart diseases and stroke were also based on patient’s response to the self-reported questionnaire, so we were not able to discern whether stroke indicates ischemic or hemorrhagic and whether heart diseases means ischemic heart diseases or others. Thus, there could be a possibility of over-adjustment owing to overlap of various conditions in covariates. Lastly, as mentioned earlier, the timing and dosage of HRT could not be validated in the present study, and this necessitates future research with more detailed information on HRT.

In conclusion, this nationwide population-based health screening cohort study investigated the association between female reproductive parameters and the risk of retinal vascular occlusions and revealed that early menopause is an independent risk factor for future RVO and RAO development. Our findings suggest that clinicians should obtain reproductive history when dealing with postmenopausal women with a possible risk of retinal vascular occlusions and inform patients with early menopause that they have a higher risk of retinal vascular occlusion. Future research is warranted to establish clear evidence of the effect of HRT on the risk of retinal vascular occlusions.

## Supplementary Information

Supplementary Tables.

## Data Availability

The datasets analyzed in the current study were provided by the Korean NHIS. The data are available at https://nhiss.nhis.or.kr with the permission of the NHIS.

## References

[1] Ehlers JP, Fekrat S (2011). Retinal vein occlusion: Beyond the acute event. Surv. Ophthalmol..

[2] Hayreh SS (2011). Acute retinal arterial occlusive disorders. Prog. Retin Eye Res..

[3] Klein R, Moss SE, Meuer SM, Klein BE (2008). The 15-year cumulative incidence of retinal vein occlusion: The Beaver Dam Eye Study. Arch. Ophthalmol..

[4] Leavitt, J. A., Larson, T. A., Hodge, D. O. & Gullerud, R. E. The incidence of central retinal artery occlusion in Olmsted County, Minnesota. *Am. J. Ophthalmol.* **152**, 820-823 e822 (2011). 10.1016/j.ajo.2011.05.005 PMC3326414 21794842

[5] Song P, Xu Y, Zha M, Zhang Y, Rudan I (2019). Global epidemiology of retinal vein occlusion: A systematic review and meta-analysis of prevalence, incidence, and risk factors. J. Glob. Health.

[6] Park SJ, Choi NK, Park KH, Woo SJ (2014). Nationwide incidence of clinically diagnosed retinal vein occlusion in Korea, 2008 through 2011: Preponderance of women and the impact of aging. Ophthalmology.

[7] Park SJ, Choi NK, Seo KH, Park KH, Woo SJ (2014). Nationwide incidence of clinically diagnosed central retinal artery occlusion in Korea, 2008 to 2011. Ophthalmology.

[8] Zhao J, Sastry SM, Sperduto RD, Chew EY, Remaley NA (1993). Arteriovenous crossing patterns in branch retinal vein occlusion. The Eye Disease Case-Control Study Group. Ophthalmology.

[9] Christoffersen NL, Larsen M (1999). Pathophysiology and hemodynamics of branch retinal vein occlusion. Ophthalmology.

[10] Kumar B (1998). The distribution of angioarchitectural changes within the vicinity of the arteriovenous crossing in branch retinal vein occlusion. Ophthalmology.

[11] Yasuda M (2010). Prevalence and systemic risk factors for retinal vein occlusion in a general Japanese population: The Hisayama study. Invest. Ophthalmol. Vis. Sci..

[12] Hayreh SS, Podhajsky PA, Zimmerman MB (2009). Retinal artery occlusion: Associated systemic and ophthalmic abnormalities. Ophthalmology.

[13] Scott IU, Campochiaro PA, Newman NJ, Biousse V (2020). Retinal vascular occlusions. Lancet.

[14] Zhu D (2019). Age at natural menopause and risk of incident cardiovascular disease: A pooled analysis of individual patient data. Lancet Public Health.

[15] Parikh NI (2016). Reproductive risk factors and coronary heart disease in the women's health initiative observational study. Circulation.

[16] Seong SC (2017). Cohort profile: The National Health Insurance Service-National Health Screening Cohort (NHIS-HEALS) in Korea. BMJ Open.

[17] Kim Y, Jun JK, Choi KS, Lee HY, Park EC (2011). Overview of the National Cancer screening programme and the cancer screening status in Korea. Asian Pac. J. Cancer Prev..

[18] Jeon KH (2020). Female reproductive factors and the risk of lung cancer in postmenopausal women: A nationwide cohort study. Br. J. Cancer.

[19] Yoo JE (2021). Association of female reproductive factors with incidence of fracture among postmenopausal women in Korea. JAMA Netw. Open.

[20] Yoo JE (2020). Female reproductive factors and the risk of Parkinson's disease: A nationwide cohort study. Eur. J. Epidemiol..

[21] World Health Organization. Regional Office for the Western, P. *The Asia-Pacific perspective: redefining obesity and its treatment*. 55p. (Sydney : Health Communications Australia, 2000).

[22] Klein R, Klein BE, Moss SE, Meuer SM (2003). Retinal emboli and cardiovascular disease: The Beaver Dam Eye Study. Arch. Ophthalmol..

[23] Cugati S, Wang JJ, Rochtchina E, Mitchell P (2006). Ten-year incidence of retinal vein occlusion in an older population: The Blue Mountains Eye Study. Arch. Ophthalmol..

[24] Cugati S, Wang JJ, Rochtchina E, Mitchell P (2006). Ten-year incidence of retinal emboli in an older population. Stroke.

[25] Santoro N (2003). Mechanisms of premature ovarian failure. Ann. Endocrinol. (Paris).

[26] Shifren JL, Gass ML (2014). The North American Menopause Society recommendations for clinical care of midlife women. Menopause.

[27] Muka T (2016). Association of age at onset of menopause and time since onset of menopause with cardiovascular outcomes, intermediate vascular traits, and all-cause mortality: A systematic review and meta-analysis. JAMA Cardiol..

[28] Mendelsohn ME, Karas RH (1999). The protective effects of estrogen on the cardiovascular system. N. Engl. J. Med..

[29] Knowlton AA, Lee AR (2012). Estrogen and the cardiovascular system. Pharmacol. Ther..

[30] Xing D, Nozell S, Chen YF, Hage F, Oparil S (2009). Estrogen and mechanisms of vascular protection. Arterioscler. Thromb. Vasc. Biol..

[31] Manson JE (2013). Menopausal hormone therapy and health outcomes during the intervention and extended poststopping phases of the Women's Health Initiative randomized trials. JAMA.

[32] Grady D (2002). Cardiovascular disease outcomes during 6.8 years of hormone therapy: Heart and Estrogen/progestin Replacement Study follow-up (HERS II). JAMA.

[33] Manson JE (2003). Estrogen plus progestin and the risk of coronary heart disease. N. Engl. J. Med..

[34] Rossouw JE (2007). Postmenopausal hormone therapy and risk of cardiovascular disease by age and years since menopause. JAMA.

[35] Grodstein F, Clarkson TB, Manson JE (2003). Understanding the divergent data on postmenopausal hormone therapy. N. Engl. J. Med..

[36] Lee SR (2020). The 2020 menopausal hormone therapy guidelines. J. Menopausal Med..

[37] de Villiers TJ (2016). Revised global consensus statement on menopausal hormone therapy. Maturitas.

[38] Zhang X (2013). Socioeconomic disparity in use of eye care services among US adults with age-related eye diseases: National Health Interview Survey, 2002 and 2008. JAMA Ophthalmol..

[39] Roerecke M, Rehm J (2014). Alcohol consumption, drinking patterns, and ischemic heart disease: A narrative review of meta-analyses and a systematic review and meta-analysis of the impact of heavy drinking occasions on risk for moderate drinkers. BMC Med..

[40] Piano MR (2017). Alcohol's effects on the cardiovascular system. Alcohol. Res..

[41] Hines LM, Rimm EB (2001). Moderate alcohol consumption and coronary heart disease: A review. Postgrad. Med. J..

[42] The Eye Disease Case-control Study Group (1993). Risk factors for branch retinal vein occlusion. Am. J. Ophthalmol..

[43] The Eye Disease Case-Control Study Group (1996). Risk factors for central retinal vein occlusion. Arch. Ophthalmol..

