# Supplementary material for: Early menopause is associated with increased risk of retinal vascular occlusions: a nationwide cohort study
Source: Sci Rep. 2022 Apr 12;12:6068. doi: 10.1038/s41598-022-10088-0 (PMC9005535; doi:10.1038/s41598-022-10088-0)
Supplement: Supplementary file 1 — Supplementary Tables. [file 41598_2022_10088_MOESM1_ESM.pdf]

Early menopause is associated with increased risk of retinal vascular occlusions: a nationwide cohort study

Sungsoon Hwang, MD[1,2]; Se Woong Kang, MD, PhD[1]; Kyung Jun Choi, MD[3]; Ki Young Son, MD[1]; Dong Hui Lim, MD, PhD[1,2]; Dong Wook Shin, MD, PhD[2,4]; DooSeok Choi, MD, PhD[5]; Sang Jin Kim, MD, PhD[1]

[1]Department of Ophthalmology, Samsung Medical Center, Sungkyunkwan University School of Medicine, Seoul, Republic of Korea

[2]Department of Clinical Research Design & Evaluation, Samsung Advanced Institute for Health Sciences and Technology (SAIHST), Sungkyunkwan University, Seoul, Republic of Korea

[3]Department of Ophthalmology, Gangneung Asan Hospital, University of Ulsan College of Medicine, Gangneung, Republic of Korea

[4]Department of Family Medicine and Supportive Care Center, Samsung Medical Center, Sungkyunkwan University School of Medicine, Seoul, Republic of Korea

[5]Department of Obstetrics and Gynecology, Samsung Medical Center, Sungkyunkwan University School of Medicine, Seoul, Republic of Korea

Corresponding Author:

Se Woong Kang, MD, PhD

Department of Ophthalmology, Samsung Medical Center, Sungkyunkwan University School of Medicine, #81 Irwon-ro, Gangnam-gu, Seoul 06351, Republic of Korea

Tel: 82-2-3410-3548, Fax: 82-2-3410-0074, E-mail: kangsewoong@gmail.com

Word count: 3,206

Short Title: Reproductive factors and retinal vascular occlusions

Keywords: early menopause; estrogen; hormone replacement therapy; reproductive factors; retinal artery occlusion; retinal vein occlusion

Supplemental Table 1. Baseline characteristics of the study population with and without retinal vein occlusion and retinal artery occlusion.

Variables	Retinal Vein Occlusion		Retinal Artery Occlusion	
	No<br>(N = 2,281,886)	Yes<br>(N = 7,461)	No<br>(N = 2,287,744)	Yes<br>(N = 1,603)
1. Demographic Factors				
Age, years, mean ± SD	62.40 ± 8.25	65.49 ± 8.11	62.40 ± 8.25	66.57 ± 8.23
Age group, No. (%)				
50–54 years	470,714 (20.63)	758 (10.16)	471,319 (20.60)	153 (9.54)
55–59 years	495,580 (21.72)	1,141 (15.29)	496,546 (21.70)	175 (10.92)
60–64 years	512,873 (22.48)	1,712 (22.95)	514,230 (22.48)	355 (22.15)
65–69 years	290,477 (12.73)	1,241 (16.63)	291,437 (12.74)	281 (17.53)
70–74 years	309,162 (13.55)	1,590 (21.31)	310,388 (13.57)	364 (22.71)
75–79 years	125,302 (5.49)	651 (8.73)	125,781 (5.50)	172 (10.73)
≥ 80 years	77,778 (3.41)	368 (4.93)	78,043 (3.41)	103 (6.43)
Income, No. (%)				
Q1 (lowest)	518,569 (22.73)	1,726 (23.13)	519,929 (22.73)	366 (22.83)
Q2	403,342 (17.68)	1,178 (15.79)	404,282 (17.67)	238 (14.85)
Q3	538,797 (23.61)	1,729 (23.17)	540,157 (23.61)	369 (23.02)
Q4 (highest)	821,178 (35.99)	2,828 (37.90)	823,376 (35.99)	630 (39.30)
2. Systemic Comorbidities				
Hypertension, No. (%)				
No	1,327,810 (58.19)	3,391 (45.45)	1,330,542 (58.16)	659 (41.11)
Yes	954,076 (41.81)	4,070 (54.55)	957,202 (41.84)	944 (58.89)
Diabetes mellitus, No. (%)				
No	1,949,881 (85.45)	5,424 (72.7)	1,954,188 (85.42)	1,117 (69.68)
Yes	332,005 (14.55)	2,037 (27.3)	333,556 (14.58)	486 (30.32)
Dyslipidemia, No. (%)				
No	1,619,561 (70.97)	4,527 (60.68)	1,623,169 (70.95)	919 (57.33)
Yes	662,325 (29.03)	2,934 (39.32)	664,575 (29.05)	684 (42.67)
Stroke, No. (%)				
No	2,245,771 (98.42)	7,278 (97.55)	2,251,498 (98.42)	1,551 (96.76)
Yes	36,115 (1.58)	183 (2.45)	36,246 (1.58)	52 (3.24)
Heart diseases, No. (%)				
No	2,189,588 (95.96)	6,974 (93.47)	2,195,110 (95.95)	1,452 (90.58)
Yes	92,298 (4.04)	487 (6.53)	92,634 (4.05)	151 (9.42)
Chronic kidney disease, No. (%)				
No	2,093,755 (91.76)	6,454 (86.50)	2,098,820 (91.74)	1,389 (86.65)
Yes	188,131 (8.24)	1,007 (13.50)	188,924 (8.26)	214 (13.35)
3. Behavioral Factors				
Smoking history, No. (%)				
Never smoked	2,203,471 (96.56)	7,203 (96.54)	2,209,129 (96.56)	1,545 (96.38)
Former smoker	25,877 (1.13)	94 (1.26)	25,947 (1.13)	24 (1.50)
Current smoker	52,538 (2.30)	164 (2.20)	52,668 (2.30)	34 (2.12)
Drinking habit, No. (%)				
None	1,980,981 (86.81)	6,803 (91.18)	1,986,303 (86.82)	1,481 (92.39)
Mild	282,979 (12.40)	610 (8.18)	283,474 (12.39)	115 (7.17)
Heavy	17,926 (0.79)	48 (0.64)	17,967 (0.79)	7 (0.44)
Regular physical activity, No. (%)				
No	1,790,709 (78.47)	5,929 (79.47)	492,384 (21.52)	325 (20.27)
Yes	491,177 (21.53)	1,532 (20.53)	492,384 (21.52)	325 (20.27)
Body mass index, No (%)				
< 18.5 kg/m^2	54,063 (2.37)	162 (2.17)	54,197 (2.37)	28 (1.75)
18.5 to < 23 kg/m^2	838,424 (36.74)	2,520 (33.78)	840,386 (36.73)	558 (34.81)
23 to < 25 kg/m^2	588,436 (25.79)	1,912 (25.63)	589,931 (25.79)	417 (26.01)
25 to < 30 kg/m^2	702,889 (30.80)	2,524 (33.83)	704,887 (30.81)	526 (32.81)
≥ 30 kg/m^2	98,074 (4.30)	343 (4.60)	98,343 (4.30)	74 (4.62)
4. Reproductive Factors				
Age at menarche, mean ± SD	16.19 ± 2.02	16.43 ± 2.02	16.19 ± 2.02	16.39 ± 1.90

Age at menarche in group, No. (%)				
< 14 years	142,233 (6.23)	344 (4.61)	142,490 (6.23)	87 (5.43)
14–15 years	732,015 (32.08)	2,151 (28.83)	733,731 (32.07)	435 (27.14)
16–17 years	872,451 (38.23)	2,891 (38.75)	874,691 (38.23)	651 (40.61)
≥ 18 years	535,187 (23.45)	2,075 (27.81)	536,832 (23.47)	430 (26.82)
Age at menopause, mean ± SD	50.63 ± 3.91	50.40 ± 4.20	50.63 ± 3.91	50.25 ± 4.25
Age at menopause in group, No. (%)				
< 45 years	120,340 (5.27)	534 (7.16)	120,758 (5.28)	116 (7.24)
45–49 years	528,004 (23.14)	1,782 (23.88)	529,377 (23.14)	409 (25.51)
50–54 years	1,329,533 (58.26)	4,098 (54.93)	1,332,762 (58.26)	869 (54.21)
≥ 55 years	304,009 (13.32)	1,047 (14.03)	304,847 (13.33)	209 (13.04)
Parity, No. (%)				
Nulliparous	40,081 (1.76)	130 (1.74)	40,196 (1.76)	15 (0.94)
1 child	185,738 (8.14)	518 (6.94)	186,155 (8.14)	101 (6.30)
≥ 2 children	2,056,067 (90.1)	6,813 (91.31)	2,061,393 (90.11)	1,487 (92.76)
Duration of breastfeeding, No. (%)				
Never	190,885 (8.37)	528 (7.08)	191,323 (8.36)	90 (5.61)
< 0.5 year	208,328 (9.13)	488 (6.54)	208,702 (9.12)	114 (7.11)
0.5 to < 1 year	425,305 (18.64)	1,123 (15.05)	426,158 (18.63)	270 (16.84)
≥ 1 year	1,457,368 (63.87)	5,322 (71.33)	1,461,561 (63.89)	1,129 (70.43)
Hormone replacement therapy, No. (%)				
Never used	1,828,479 (80.13)	5,995 (80.35)	1,833,180 (80.13)	1,294 (80.72)
< 2 years	202,176 (8.86)	620 (8.31)	202,676 (8.86)	120 (7.49)
2 to < 5 years	85,995 (3.77)	286 (3.83)	86,216 (3.77)	65 (4.05)
≥ 5 years	70,313 (3.08)	266 (3.57)	70,528 (3.08)	51 (3.18)
Unknown	94,923 (4.16)	294 (3.94)	95,144 (4.16)	73 (4.55)
Oral contraceptive pill use, No. (%)				
Never used	1,832,942 (80.33)	5,919 (79.33)	1,837,574 (80.32)	1,287 (80.29)
< 1 year	201,039 (8.81)	682 (9.14)	201,590 (8.81)	131 (8.17)
≥ 1 year	134,160 (5.88)	501 (6.71)	134,558 (5.88)	103 (6.43)
Unknown	113,745 (4.98)	359 (4.81)	114,022 (4.98)	82 (5.12)

SD, standard deviation; Q, quartile

Supplemental Table 2. Hazard ratios and 95% confidence intervals for development of retinal vein occlusion in postmenopausal women according to covariates.

	Subject No.	Case No.	Duration (person-years)	IR per 100,000 person-years	Model 1 HR (95% CI)	Model 2 HR (95% CI)	Model 3 HR (95% CI)
Overall	2,289,347	7,461	11,192,885	66.66			
1. Demographic Factors							
Age					1.04 (1.04–1.05)	1.03 (1.03–1.04)	1.03 (1.03–1.03)
Income							
Q1 (lowest)	520,295	1,726	2,553,456	67.59	1.00 (ref)	1.00 (ref)	1.00 (ref)
Q2	404,520	1,178	1,980,613	59.48	0.94 (0.87–1.00)	0.94 (0.88–1.01)	0.94 (0.88–1.01)
Q3	540,526	1,729	2,646,734	65.33	0.97 (0.91–1.03)	0.97 (0.91–1.04)	0.97 (0.91–1.04)
Q4 (highest)	824,006	2,828	4,012,082	70.49	0.95 (0.90–1.01)	0.96 (0.91–1.02)	0.96 (0.91–1.02)
2. Systemic Comorbidities							
Hypertension							
No	1,331,201	3,391	6,513,358	52.06	1.00 (ref)	1.00 (ref)	1.00 (ref)
Yes	958,146	4,070	4,679,528	86.97	1.34 (1.27–1.40)	1.14 (1.08–1.20)	1.14 (1.08–1.20)
Diabetes mellitus							
No	1,955,305	5,424	9,570,393	56.68	1.00 (ref)	1.00 (ref)	1.00 (ref)
Yes	334,042	2,037	1,622,492	125.55	1.90 (1.80–2.00)	1.70 (1.61–1.80)	1.70 (1.61–1.80)
Dyslipidemia							
No	1,624,088	4,527	7,946,274	56.97	1.00 (ref)	1.00 (ref)	1.00 (ref)
Yes	665,259	2,934	3,246,612	90.37	1.42 (1.36–1.49)	1.15 (1.09–1.21)	1.15 (1.09–1.21)
Stroke							
No	2,253,049	7,278	11,017,933	66.06	1.00 (ref)	1.00 (ref)	1.00 (ref)
Yes	36,298	183	174,952	104.60	1.25 (1.08–1.45)	1.09 (0.94–1.26)	1.09 (0.94–1.26)
Heart disease							
No	2,196,562	6,974	10,743,063	64.92	1.00 (ref)	1.00 (ref)	1.00 (ref)
Yes	92,785	487	449,822	108.27	1.31 (1.19–1.43)	1.10 (1.00–1.21)	1.09 (1.00–1.20)
Chronic kidney disease							
No	2,100,209	6,454	10,277,203	62.80	1.00 (ref)	1.00 (ref)	1.00 (ref)
Yes	189,138	1,007	915,683	109.97	1.31 (1.23–1.41)	1.20 (1.12–1.28)	1.20 (1.12–1.28)
3. Behavioral Factors							
Smoking history							
Never smoked	2,210,674	7,203	10,811,786	66.62	1.00 (ref)	1.00 (ref)	1.00 (ref)
Former smoker	25,971	94	125,375	74.98	1.20 (0.98–1.47)	1.19 (0.97–1.46)	1.18 (0.96–1.45)
Current smoker	52,702	164	255,724	64.13	1.08 (0.92–1.26)	1.09 (0.93–1.28)	1.07 (0.92–1.26)

Drinking habit							
None	1,987,784	6,803	9,721,987	69.98	1.00 (ref)	1.00 (ref)	1.00 (ref)
Mild	283,589	610	1,383,139	44.10	0.77 (0.71–0.84)	0.79 (0.73–0.86)	0.79 (0.72–0.86)
Heavy	17,974	48	87,760	54.69	0.87 (0.66–1.16)	0.90 (0.68–1.19)	0.89 (0.67–1.18)
Regular physical activity							
No	1,796,638	5,929	8,780,732	67.52	1.00 (ref)	1.00 (ref)	1.00 (ref)
Yes	492,709	1,532	2,412,154	63.51	1.02 (0.96–1.08)	1.02 (0.96–1.08)	1.02 (0.96–1.08)
Body mass index							
< 18.5 kg/m^2	54,225	162	257,462	62.92	0.91 (0.77–1.06)	0.96 (0.81–1.12)	0.96 (0.82–1.12)
18.5 to < 23 kg/m^2	840,944	2,520	4,102,525	61.43	1.00 (ref)	1.00 (ref)	1.00 (ref)
23 to < 25 kg/m^2	590,348	1,912	2,893,499	66.08	1.04 (0.98–1.11)	1.00 (0.95–1.07)	1.00 (0.94–1.06)
25 to < 30 kg/m^2	705,413	2,524	3,458,875	72.97	1.11 (1.05–1.17)	1.02 (0.96–1.08)	1.02 (0.96–1.08)
≥ 30 kg/m^2	98,417	343	480,524	71.38	1.09 (0.98–1.23)	0.91 (0.82–1.03)	0.92 (0.82–1.03)

IR, incidence rate; HR, hazard ratio; CI, confidence interval; Q, quartile

Model 1: adjusted for age.

Model 2: adjusted for age, income level, systemic comorbidities (hypertension, diabetes mellitus, dyslipidemia, stroke, heart disease, and chronic kidney disease), and behavioral factors (smoking history, drinking habit, physical activity, body mass index).

Model 3: adjusted for age, income level, systemic comorbidities, behavioral factors, and female reproductive factors (age at menarche, age at menopause, parity, breastfeeding, hormone replacement therapy, oral contraceptive pill).

Supplemental Table 3. Hazard ratios and 95% confidence intervals for development of retinal artery occlusion in postmenopausal women according to covariates.

	Subject No.	Case No.	Duration (person-years)	IR per 100,000 person-years	Model 1 HR (95% CI)	Model 2 HR (95% CI)	Model 3 HR (95% CI)
Overall	2,289,347	1,603	11,207,750	14.30			
1. Demographic Factors							
Age					1.06 (1.05–1.06)	1.04 (1.04–1.05)	1.04 (1.04–1.05)
Income							
Q1 (lowest)	520,295	366	2,556,893	14.31	1.00 (ref)	1.00 (ref)	1.00 (ref)
Q2	404,520	238	1,982,930	12.00	0.89 (0.76–1.04)	0.90 (0.77–1.05)	0.89 (0.76–1.04)
Q3	540,526	369	2,650,170	13.92	0.96 (0.84–1.10)	0.96 (0.84–1.11)	0.96 (0.84–1.10)
Q4 (highest)	824,006	630	4,017,758	15.68	0.95 (0.84–1.08)	0.95 (0.84–1.08)	0.95 (0.84–1.08)
2. Systemic Comorbidities							
Hypertension							
No	1,331,201	659	6,520,108	10.11	1.00 (ref)	1.00 (ref)	1.00 (ref)
Yes	958,146	944	4,687,642	20.14	1.48 (1.33–1.65)	1.22 (1.09–1.37)	1.23 (1.09–1.38)
Diabetes mellitus							
No	1,955,305	1,117	9,581,180	11.66	1.00 (ref)	1.00 (ref)	1.00 (ref)
Yes	334,042	486	1,626,570	29.88	2.09 (1.87–2.32)	1.80 (1.60–2.03)	1.81 (1.61–2.03)
Dyslipidemia							
No	1,624,088	919	7,955,344	11.55	1.00 (ref)	1.00 (ref)	1.00 (ref)
Yes	665,259	684	3,252,406	21.03	1.58 (1.43–1.74)	1.20 (1.07–1.34)	1.20 (1.07–1.34)
Stroke							
No	2,253,049	1,551	11,032,451	14.06	1.00 (ref)	1.00 (ref)	1.00 (ref)
Yes	36,298	52	175,299	29.66	1.57 (1.19–2.07)	1.31 (0.99–1.73)	1.31 (0.99–1.73)
Heart disease							
No	2,196,562	1,452	10,757,042	13.50	1.00 (ref)	1.00 (ref)	1.00 (ref)
Yes	92,785	151	450,709	33.50	1.82 (1.53–2.16)	1.49 (1.25–1.77)	1.48 (1.25–1.77)
Chronic kidney disease							
No	2,100,209	1,389	10,289,892	13.50	1.00 (ref)	1.00 (ref)	1.00 (ref)
Yes	189,138	214	917,858	23.32	1.17 (1.01–1.36)	1.02 (0.88–1.19)	1.02 (0.88–1.19)
3. Behavioral Factors							
Smoking history							
Never smoked	2,210,674	1,545	10,826,139	14.27	1.00 (ref)	1.00 (ref)	1.00 (ref)
Former smoker	25,971	24	125,557	19.11	1.45 (0.97–2.17)	1.43 (0.95–2.14)	1.44 (0.96–2.16)
Current smoker	52,702	34	256,055	13.28	1.08 (0.77–1.52)	1.13 (0.80–1.59)	1.13 (0.80–1.60)

Drinking habit							
None	1,987,784	1,481	9,735,550	15.21	1.00 (ref)	1.00 (ref)	1.00 (ref)
Mild	283,589	115	1,384,332	8.31	0.73 (0.60–0.88)	0.75 (0.61–0.91)	0.74 (0.61–0.90)
Heavy	17,974	7	87,868	7.97	0.61 (0.29–1.27)	0.62 (0.30–1.31)	0.62 (0.30–1.31)
Regular physical activity							
No	1,796,638	1,278	8,792,518	14.54	1.00 (ref)	1.00 (ref)	1.00 (ref)
Yes	492,709	325	2,415,232	13.46	1.04 (0.92–1.17)	1.03 (0.91–1.17)	1.03 (0.91–1.17)
Body mass index							
< 18.5 kg/m^2	54,225	28	257,825	10.86	0.67 (0.46–0.99)	0.73 (0.50–1.07)	0.73 (0.50–1.07)
18.5 to < 23 kg/m^2	840,944	558	4,107,491	13.58	1.00 (ref)	1.00 (ref)	1.00 (ref)
23 to < 25 kg/m^2	590,348	417	2,897,292	14.39	1.02 (0.90–1.16)	0.97 (0.85–1.10)	0.97 (0.86–1.11)
25 to < 30 kg/m^2	705,413	526	3,463,987	15.18	1.03 (0.92–1.16)	0.92 (0.81–1.04)	0.93 (0.82–1.05)
≥ 30 kg/m^2	98,417	74	481,154	15.38	1.06 (0.83–1.35)	0.84 (0.66–1.08)	0.85 (0.67–1.09)

IR, incidence rate; HR, hazard ratio; CI, confidence interval; Q, quartile

Model 1: adjusted for age.

Model 2: adjusted for age, income level, systemic comorbidities (hypertension, diabetes mellitus, dyslipidemia, stroke, heart disease, and chronic kidney disease), and behavioral factors (smoking history, drinking habit, physical activity, body mass index).

Model 3: adjusted for age, income level, systemic comorbidities, behavioral factors, and female reproductive factors (age at menarche, age at menopause, parity, breastfeeding, hormone replacement therapy, oral contraceptive pill).

Supplemental Table 4. Hazard ratios and 95% confidence intervals for development of retinal vein occlusion and retinal artery occlusion in postmenopausal women according to history of hormone replacement therapy.

	**Age at menopause**			
	< 45 years	45–49 years	50–54 years	≥ 55 years
Full-Adjusted (Model 3)	HR (95% CI)	HR (95% CI)	HR (95% CI)	HR (95% CI)
Retinal Vein Occlusion				
Hormone replacement therapy				
Never used	1.00 (ref)	1.00 (ref)	1.00 (ref)	1.00 (ref)
< 2 years	0.91 (0.64–1.29)	1.02 (0.86–1.21)	1.11 (0.99–1.24)	1.08 (0.86–1.35)
2–5 years	0.95 (0.57–1.57)	1.07 (0.84–1.36)	1.24 (0.99–1.53)	1.10 (0.80–1.51)
≥ 5 years	1.00 (0.66–1.52)	1.26 (0.98–1.61)	1.09 (0.90–1.32)	1.34 (0.99–1.83)
Unknown	1.05 (0.67–1.67)	0.83 (0.63–1.10)	0.92 (0.78–1.09)	1.09 (0.79–1.51)
Retinal Artery Occlusion				
Hormone replacement therapy				
Never used	1.00 (ref)	1.00 (ref)	1.00 (ref)	1.00 (ref)
< 2 years	1.38 (0.70–2.71)	0.97 (0.68–1.40)	0.90 (0.68–1.19)	1.30 (0.81–2.07)
2–5 years	1.59 (0.63–3.99)	1.36 (0.87–2.13)	1.32 (0.94–1.87)	0.72 (0.30–1.76)
≥ 5 years	1.51 (0.68–3.35)	0.77 (0.43–1.37)	0.94 (0.60–1.47)	1.99 (0.95–3.90)
Unknown	0.68 (0.21–2.24)	0.96 (0.56–1.66)	1.09 (0.78–1.51)	1.19 (0.59–2.40)
	**Age at baseline**			
	50-59 years	60–69 years	70–79 years	≥ 80 years
Full-Adjusted (Model 3)	HR (95% CI)	HR (95% CI)	HR (95% CI)	HR (95% CI)
Retinal Vein Occlusion				
Hormone replacement therapy				
Never used	1.00 (ref)	1.00 (ref)	1.00 (ref)	1.00 (ref)
< 2 years	1.09 (0.94–1.26)	1.09 (0.96–1.23)	0.90 (0.73–1.10)	1.97 (0.95–3.88)
2–5 years	1.29 (0.97–1.68)	1.02 (0.86–1.22)	1.17 (0.89–1.54)	0.81 (0.20–3.26)
≥ 5 years	1.16 (0.88–1.54)	1.02 (0.86–1.21)	1.34 (0.97–1.80)	0.46 (0.07–3.32)
Unknown	0.87 (0.66–1.14)	0.99 (0.81–1.21)	0.99 (0.80–1.24)	0.70 (0.42–1.15)
Retinal Artery Occlusion				
Hormone replacement therapy				
Never used	1.00 (ref)	1.00 (ref)	1.00 (ref)	1.00 (ref)
< 2 years	1.11 (0.78–1.58)	0.97 (0.74–1.28)	0.79 (0.51–1.23)	2.60 (0.94–7.19)
2–5 years	1.65 (0.97–2.71)	1.15 (0.80–1.65)	1.01 (0.55–1.84)	1.52 (0.21–11.0)
≥ 5 years	1.14 (0.56–2.33)	1.01 (0.70–1.47)	0.92 (0.52–1.64)	1.83 (0.25–13.3)
Unknown	1.62 (0.97–2.71)	0.78 (0.49–1.26)	0.95 (0.61–1.45)	1.62 (0.73–3.60)
